# Transcriptional insights into aflatoxin B1 induced hepatotoxicity and comparative effects of medicinal herbs in pigs

**DOI:** 10.1186/s12917-025-05270-1

**Published:** 2026-01-15

**Authors:** Avon Augustin Nalpadan, Henry Reyer, Michael Oster, Nares Trakooljul, Siriluck Ponsuksili, Wojciech Kozera, Krzysztof Karpiesiuk, Katarzyna Kępka-Borkowska, Katarzyna Chałaśkiewicz, Mariusz Pierzchała, Hiroaki Taniguchi, Adam Lepczyński, Brygida Ślaska, Varunkumar Asediya, Chandra Shekhar Pareek, Klaus Wimmers

**Affiliations:** 1 Research Institute for Farm Animal Biology (FBN), Wilhelm-Stahl-Allee 2, 18196 Dummerstorf, Germany; 2 Department of Pig Breeding, Faculty of Animal Bio-Engineering, University of Warmia and Mazury in Olsztyn, Michała Oczapowskiego St. 5, 10-959 Olsztyn, Poland; 3 Department of Genomics and Biodiversity, Institute of Genetics and Animal Biotechnology of the Polish Academy of Sciences, Postępu St. 36A, 05-552 Jastrzębiec, Poland; 4 African Genome Center, Mohammed VI Polytechnic University, Lot 660, Hay Moulay Rachid Ben Guerir, 43150 Ben Guerir, Morocco; 5 Faculty of Biotechnology and Animal Sciences, Department of Physiology, Cytobiology and Proteomics, West Pomeranian University of Technology in Szczecin, Janickiego 29 Str., 71-270 Szczecin, Poland; 6 Faculty of Animal Sciences and Bioeconomy, University of Life Sciences in Lublin, Akademicka 13 Str, Lublin, 20-950 Poland; 7 Institute of Veterinary Medicine, Department of Infectious, Faculty of Biological and Veterinary Sciences, Nicolaus Copernicus University, Lwowska St. 1, Toruń, 87-100 Poland; 8 Faculty of Agricultural, Civil and Environmental Engineering, University of Rostock, Justus-von-Liebig-Weg 6, 18059 Rostock, Germany

**Keywords:** Andrographolide, Biotransformation, Detoxification, Gene expression, Herb supplementation, Liver integrity, Metabolic pathways;

## Abstract

**Background:**

Aflatoxin B1 (AFB1) contamination in animal feed poses a serious risk to livestock health due to its hepatotoxic effects. Many medicinal herbs which may be used as feed additives exhibit antioxidant and anti-inflammatory properties with potential hepatoprotective outcomes. We investigated effects of AFB1 in three concentrations (30 µg/kg BW, 60 µg/kg BW, 120 µg/kg BW) as well as three medicinal herbs, i.e., kalmegh (*Andrographis paniculata*), milk thistle (*Silybum marianum*), and turmeric (*Curcuma longa*) in pigs. Hepatic expression of genes involved in biotransformation, detoxification, antioxidation, energy homeostasis, and immunity were evaluated by high-throughput real-time PCR.

**Results:**

We found that AFB1 significantly suppressed genes involved in biotransformation (*CYP2U1*, *CYP4V2*, *CYP7B1*, *CYP26A1*, *CYP51A1*), detoxification (*GSS*, *ABCC2*, *SULT1E1*), redox balance (*GPX1*, *PRDX4*), lipid homeostasis (*ACOX1*), and immune regulation (*CP*, *CRP*). Kalmegh and, to a lesser extent, milk thistle supplementation provided a comprehensive upregulation of genes involved in key hepatic pathways maintaining liver integrity. Under the specific experimental conditions, the applied dietary turmeric supplement did not induce consistent effects on the analyzed target genes.

**Conclusions:**

The results indicate that certain medicinal herbs could counteract AFB1-induced gene expression responses in liver. Their application as dietary supplements to reduce potentially harmful effects caused by AFB1 toxicity in farm animals might be an effective tool in improving animal health, productivity and food safety.

**Supplementary Information:**

The online version contains supplementary material available at 10.1186/s12917-025-05270-1.

## Background

Aflatoxins are widely present secondary metabolites produced by the fungal genera *Aspergillus* [1]. The presence of aflatoxin in agricultural products is indisputable, and the majority of potentially contaminated crops are currently used as feed for livestock [2]. The global climate change is exerting favorable conditions for fungal growth, spiking baseline aflatoxin production, and increasing the crop contamination rates [3]. Ingestion of Aflatoxin B1 (AFB1), one of the most prevalent and potent aflatoxins, can lead to aflatoxicosis resulting in acute illness or death in farm animals [4].

Elimination of toxic agents which are predominantly lipophilic substances is crucial to prevent bioaccumulation [5]. The liver utilizes biotransformation processes, in which harmful xenobiotic molecules, that cannot be scavenged or sequestered, undergo enzymatic activation in liver and then subsequent elimination through kidney or bile [6]. The biotransformation comprises two phases. Phase 1 typically involves a series of oxidations, leading to enhanced molecular reactivity [7]. Phase 2 accounts for conjugative reactions, which subsequently increase the hydrophilicity of the resulting metabolites to facilitate their excretion [8].

Toxic intermediate metabolites derived from biotransformation, if not attenuated or eliminated, can induce hepatocyte damage by impeding chemical modification of biomolecules and obstructing redox homeostasis [9]. Specifically, the metabolic processing of AFB1 in the liver leads to the formation of harmful derivatives possessing *in-vivo* genotoxicity and DNA reactivity [10]. Biotransformation by Phase 1 enzymes like CYP2A19 and CYP3A29 on AFB1 generate AFB1-exo-8,9-epoxide with mutagenic effects [11, 12]. This epoxide derivative reacts with DNA at the guanine N7 position through hydrolysis, inducing intercalation and resulting in the formation of DNA adducts [13, 14].

Herbal remedies are seen as a protective measure and are used to mitigate toxic xenobiotics [15, 16]. Medicinal herbs act as natural reservoirs of bioactive compounds with potent anti-inflammatory, anti-fibrotic, and anti-oxidant properties. Commercially available medicinal herbs such as kalmegh (*Andrographis paniculata*), milk thistle (*Silybum marianum*), and turmeric (*Curcuma longa*) possess a range of therapeutic properties and contain potentially beneficial compounds.

An in vivo phytochemical study in rats showed that bioactive compounds present in kalmegh confer therapeutic activity towards a variety of liver disorders [17]. Andrographolide, a diterpenoid lactone extracted from the whole plant of kalmegh could deliver protection against acute liver damage with its anti-inflammatory, anti-fibrotic, and immunostimulatory effects in mice [18]. Silymarin extract from milk thistle seeds was utilized as a cytoprotectant [19] and it conferred antioxidative and immunomodulatory effects in humans [20]. *In-vitro* studies utilizing flavanol constituents in silymarin indicated its contribution towards radical scavenging by modulating antioxidant enzyme activity and conferring protection against environmental contaminants [21, 22]. Spectroscopic studies involving curcumin from turmeric rhizome also confirmed its anti-oxidant effects by radical trapping mechanisms [23].

We hypothesize that the application of medicinal herbs with their specific profile of bioactive compounds could contribute to hepatic protection against toxic effects exerted by orally applied AFB1 in pigs. The objective of this study was to investigate hepatic gene expression profiles of target genes involved in biotransformation, detoxification, antioxidation, energy metabolism, and immune response using high-throughput real-time PCR by conducting two separate experiments (i) exposure to three different AFB1 levels, and (ii) administration of kalmegh, milk thistle, and turmeric supplements. This experimental separation was designed to establish baseline regulatory patterns associated with each factor individually, thereby enabling clearer interpretation of their specific effects. The findings are expected to provide mechanistic insights into AFB1-induced transcriptional response related to biotransformation in pigs and the potential hepatoprotective effects of medicinal herbs supplements for effective AFB1 clearance.

## Methods

### Dietary experiment in pigs

**Fig. 1:**
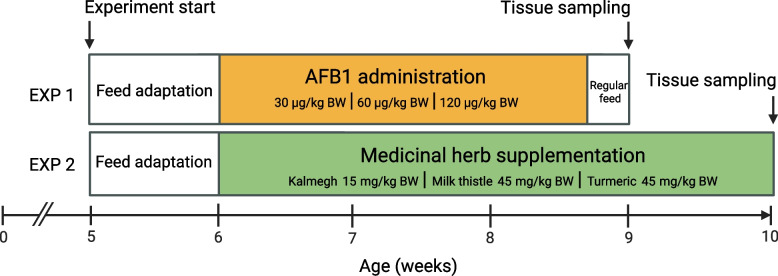
Experimental design of two individual trials on crossbred TN70 pigs. In EXP 1, a standard diet was fed until day 42, followed by aflatoxin B1 administration in 1 of 3 concentrations until day 61. In EXP 2, standard diet was fed until day 42, followed by supplementation of 1 of 3 medicinal herbs until day 70. Pigs were euthanized and slaughtered for tissue sampling at day 63 for EXP 1 and 70 for EXP 2. AFB1 – aflatoxin B1, BW – body weight

A total of 60 female commercial crossbred TN70 pigs (Norsvin Landrace x Large White) were subjected to one-week adaptation period at the age of 35 days of life (Fig. 1) and fed a standard diet (Additional file 1). Pigs were housed in pens with slatted floors and strictly monitored microclimates for ventilation (400 m^3^/ha), temperature (21 °C – 24 °C), humidity (60–70%), and artificial lighting (8 h). Water access was ad libitum via bowl waterers with running water. The nutritional study was divided into two feeding experiments (EXP). In EXP 1, a total of 24 pigs were divided into four experimental groups of six animals each. All animals were brought to the farm at the age of 35 days (mean ± SD; body weight 7.67 ± 0.81 kg) and subjected to standard feed until 42 days of age. Thereafter, AFB1 was administered through the feed at four dosage levels, i.e., control (0 µg/kg BW), low (30 µg/kg BW), medium (60 µg/kg BW), and high (120 µg/kg BW) until day 61 of life, i.e., 2 days before euthanization to ensure consistent accumulation of toxins across treatment groups. This withdrawal period was intended to assess residual detoxification outcomes resulting from the cumulative toxic burden of the 19-day dosing, without clinical signs of acute inflammation that might confound the identification of primary AFB1 effects. Aflatoxin B1 (CAS No. 1162–65-8; Item No. 11293) with a certified purity of ≥ 98% was purchased from Cayman Chemicals (Ann Arbor, MI, USA). The crystalline AFB1 was dissolved in vegetable oil for administration to ensure bioavailability according to reported toxicological assessments [24]. Doses were determined as approximately 5–30% of the reported LD50 (0.6 mg/kg BW) for pigs to ensure safety while enabling measurable effects. In EXP 2, a total of 36 pigs were allocated to three experimental groups of ten animals each, with additional 6 pigs serving as control group. All animals were subjected to standard feed until 42 days of age and then supplemented with medicinal herbs to the feed (kalmegh: 15 mg/kg BW; milk thistle: 45 mg/kg BW; turmeric: 45 mg/kg BW). Powdered extracts from Kalmegh (whole plant; andrographis content ≥ 20%), milk thistle (seed; silymarin content ≥ 80%), and turmeric (root; curcumin content ≥ 95%) were thoroughly mixed into the daily feed to achieve respective target concentrations according to the manufacturer (Swiss Herbal Institute LLC, DE, USA). Applied doses were based on the manufacturer’s recommendations for an 80 kg human and scaled to match cumulative supplement over the 4‑week pig trial. Following sedation with an intramuscular injection of azaperone (2 mg/kg BW), at the age of 63 days for EXP 1 and 70 days for EXP 2, pigs were anesthetized using an intravenous injection of Morbital plus (0.25 ml/kg BW; active substance: sodium pentobarbital) and subsequently euthanized by exsanguination. Liver samples were collected, frozen in liquid nitrogen, and stored at −80 °C until RNA extraction.

### RNA extraction from liver samples

Total RNA was extracted from EXP 1 and EXP 2 liver samples. In total, 40 mg of frozen liver sample was added into TRI Reagent (Sigma-Aldrich, Taufkirchen, Germany) and homogenized using tissue homogenizer (Precellys-24; PEQLab Biotechnology GmbH, Darmstadt, Germany). RNA extraction was performed using the phenol/chloroform method followed by DNase I digestion (Roche Diagnostics, Mannheim, Germany) and purified by NucleoSpin RNA kit (Macherey–Nagel, Düren, Germany). RNA concentrations were determined using a NanoDrop 2000 spectrophotometer (Thermo Fisher Scientific, Dreieich, Germany).

### Primer panel design and standard preparation

Through an in-depth literature review, we identified 45 target genes representing the categories biotransformation, detoxification, antioxidation, energy homeostasis and inflammation, which were used for real-time PCR-based quantification (Additional file 2). In addition, primer pairs for the specific amplification of three reference genes (*RPL32, HMBS* and *TOP2B*) were included. *TOP2B* was not considered for the later analysis due to high individual variation. For each target gene, the *Sus scrofa* primary transcript was retrieved from Ensembl and used in NCBI Primer-BLAST to generate specific primers. Primer candidates were then probed in NetPrimer (PREMIER Biosoft) for verifying optimal melting temperature (~ 60 °C), base composition (GC % > 50) and length (~ 20 bp). Primer pairs were then aligned back to the Ensembl primary assembly (Sscrofa11.1) to inspect the absence of known sequence variants in the primer sequence. In order to enable the absolute quantification of gene expression, standard curves were generated containing serial dilutions from 10^7^ to 10^2^ amplicon copies. Therefore, gene-specific amplicons were prepared with a pig liver cDNA and the LightCycler 480 SYBR Green I Master (Roche, Basel, Switzerland) on a LightCycler 480 system (Roche). Gene-specific PCR products were purified using bead purification (SPRIselect beads, Beckman Coulter, Krefeld, Germany) and concentrations were determined using a NanoDrop 2000 spectrophotometer (Thermo Fisher Scientific). Based on the predetermined amplicon size, the number of molecules per microliter in each gene-specific amplicon pool was determined using a molar quantity calculator (http://molbiol.ru/). Individual PCR products were then diluted and pooled to create a standard mix containing 10^8^ copies of each amplicon. This standard mix was subsequently used to prepare serial dilutions.

### High-throughput real-time PCR

Reverse transcription reactions of RNA samples were performed using Reverse Transcription Master Mix (Fluidigm, Munich, Germany) followed by primer pooling and multiplex PCR of cDNA (pre-amplification cycles) using PreAmp master mix (Fluidigm). The pre-amplified reaction mix was treated with exonuclease I (New England BioLabs, Ipswich, MA, USA) and loaded in the integrated fluidic circuit (IFC) as assay loading mix. The IFC used was a 96.96 format, with each sample run in technical duplicate per primer. The plate layout included assay mixes from EXP 1 and EXP 2, along with standard dilution series. The IFC was then primed to the controller HX (Fluidigm) of the BioMark HD system (Fluidigm). High-throughput real-time PCR was performed based on the system-specific protocol according to the manufacturer and the raw files were processed and mapped using Fluidigm Real-Time PCR Analysis software v4.8.1 (Fluidigm).

### Statistical analysis

All statistical analyses were performed in R Statistical Software (v4.3.2; R Core Team 2023). Thresholds of Ct < 2.5 and Ct > 30 were applied in accordance with the detection range of the Fluidigm system. Standard curves were derived from serial dilutions of the standard mix and used to generate linear regressions for further calculations. The Ct values of target and reference genes were used to derive transcript copy numbers. For each sample, the geometric mean of two reference genes (*RPL32* and *HMBS*) was used to calculate the normalized expression rate within the group. Target genes with more than 100 copies in at least ten samples per experiment were considered for downstream analysis. Copy numbers were log_2_-transformed and analyzed by one-way ANOVA. For EXP1, the AFB1 concentration was considered as a fixed effect. For EXP2, the supplementation of the distinct medicinal herb was considered as a fixed effect. Post-hoc multiple comparisons were carried out using Dunnett’s test via the multcomp package (R, version 1.4–28) to compare the treatment against the control. Statistical significance was defined as *P* < 0.05.

## Results

**Fig. 2:**
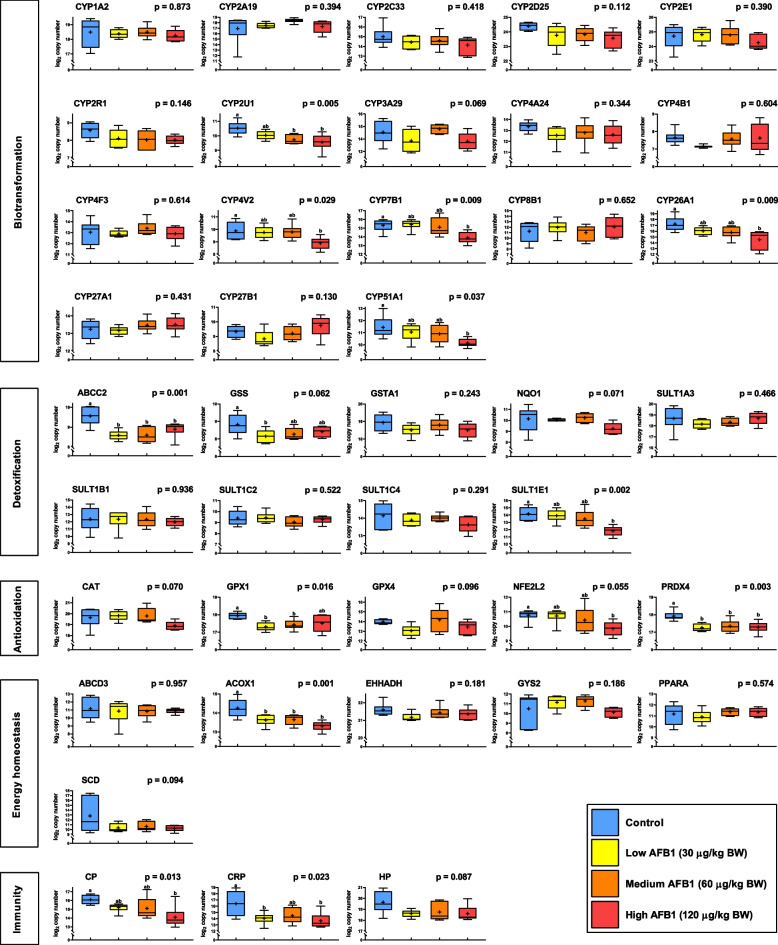
Hepatic transcript expression levels in piglets following aflatoxin B1 (AFB1) exposure (EXP 1). Animals fed a control diet (blue) are compared with groups fed diets contaminated with AFB1 at low (yellow), medium (orange), and high (red) concentrations. Functional annotations of genes are indicated. Gene names are displayed in Supplemental Table S2. Expression levels are based on log_2_-transformed copy numbers. Whiskers of boxplots represent minimal to maximal values showing all available data points. Group means are denoted by the ‘ + ’ symbol. The P-values refer to overall effects due to AFB1 exposure. Different superscripts (^a,b^) refer to significant differences between controls and AFB1-exposed groups (*P* < 0.05)

**Fig. 3:**
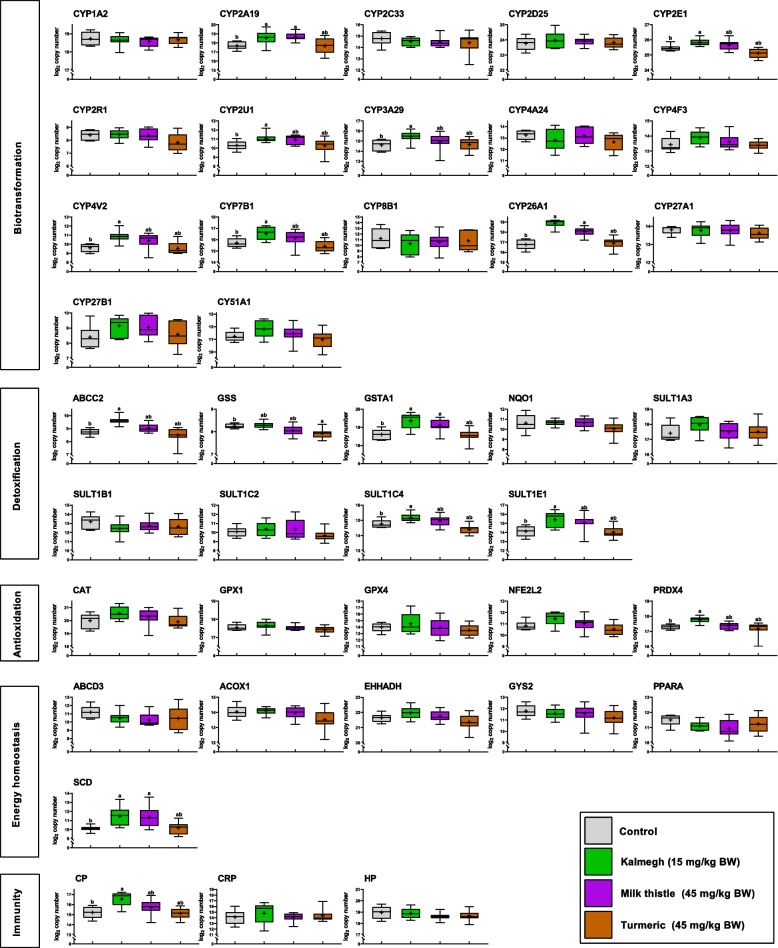
Hepatic transcript expression levels in piglets following medicinal herb supplementation (EXP 2). Animals fed a control diet (grey) are compared with groups fed diets supplemented with the medicinal herbs kalmegh (green), milk thistle (purple), and turmeric (brown). Functional annotations of genes are indicated. Gene names are displayed in Supplemental Table S2. Expression levels are based on log_2_-transformed copy numbers. Whiskers of boxplots represent minimal to maximal values showing all available data points. Group means are denoted by the ‘ + ’ symbol. Different superscripts (^a,b^) refer to significant differences between controls and individual medicinal herb-exposed groups (P < 0.05)

The biotransformation panel encompasses genes encoding for Cytochrome P450 oxidases (CYPs), which are crucial in the conversion of AFB1 into various intermediate metabolites. In EXP 1, transcript levels of *CYP2U1*, *CYP4V2*, *CYP7B1*, *CYP26A1*, and *CYP51A1* were significantly reduced in a dose-dependent manner, with the highest AFB1 concentration resulting in the lowest expression levels compared to controls. (Fig. 2). In EXP 2 dealing with medicinal herbs, *CYP2A19* and *CYP26A1* showed higher abundance in kalmegh and milk thistle groups while *CYP2E1*, *CYP2U1*, *CYP3A29*, *CYP4V2* and *CYP7B1* exclusively showed increased expression in kalmegh group compared to the controls (Fig. 3).

Among the detoxification genes that promote the reactive intermediate conjugations, *SULT1E1* showed lower abundance in the high AFB1 group compared to the control. *ABCC2* was consistently less abundant across all 3 AFB1-treated groups (Fig. 2). While a trend was observed in the ANOVA for *GSS* (*P* < 0.10), a significantly reduced expression was specifically found between the control and the low AFB1 group. Administration of medicinal herbs induced higher expression of *ABCC2*, *SULT1C4* and *SULT1E1* in the kalmegh group, while *GSTA1* expression increased in both the kalmegh and milk thistle groups compared with controls (Fig. 3). Interestingly, the turmeric administration significantly lowered the transcript abundance of *GSS* compared to control.

The antioxidant panel comprises proteins that neutralize reactive oxygen species and protect the cell from oxidative damage. Of the genes analyzed in EXP 1 following variable AFB1 exposure, *NFE2L2* showed a trend (*P* < 0.10) with a significantly reduced expression in the AFB1 high group compared to the unexposed group. *PRDX4* showed reduced abundance in all AFB1 treated groups while *GPX1* only exhibited reduced abundance in low and medium AFB1 groups (Fig. 2). Referring to the administration of medicinal herbs, EXP 2 indicated higher abundance of *PRDX4* in the kalmegh group relative to the control (Fig. 3).

The energy homeostasis functional theme included gene encoding products that play a key role in maintaining cellular energy equilibrium. Among those, *ACOX1* exhibited lower abundance in all AFB1-treated groups compared to the control in EXP 1 (Fig. 2). In EXP 2, *SCD* showed higher abundance in kalmegh and milk thistle groups as compared to the control (Fig. 3).

The immunity panel comprised genes related to host innate immunity. In EXP 1, *CP* and *CRP* exhibited statically significant differences in response to AFB1 exposure (Fig. 2). Compared to controls, *CRP* expression levels were lower in both low AFB1 and high AFB1 groups, whereas *CP* decreased only in the high AFB1 group. In EXP 2, *CP* showed higher abundance in kalmegh group compared to controls (Fig. 3).

## Discussion

Aflatoxin exposure disrupts the normal hepatic functions by modulating biotransformation and detoxification pathways. Medicinal herbs in general exhibit potential for mitigating toxin-induced damage in the liver. To dissect the hepatic molecular responses to both toxic insult and protective intervention, two independent pig trials were conducted. The first focused on dietary AFB1 exposure, while the second examined the effects of medicinal herb supplementation (kalmegh, milk thistle, and turmeric) in the absence of AFB1. This experimental separation was devised to establish baseline regulatory patterns associated with each factor individually. Liver samples were subsequently analyzed at the gene expression level to identify regulatory patterns and evaluate their functional relevance in hepatic activity. The functional classifications outlined in the results capture the key genes involved in the hepatic responses, reflecting the coordinated molecular processes underlying xenobiotic metabolism or liver adaptation. Results provide the basis to determine hepatic baseline responses to AFB1 intoxication and to establish effective supplement concentrations of medicinal herbs.

### AFB1-driven gene expression in liver

AFB1 is metabolized into several products, including aflatoxin M1 (AFM1), aflatoxin P1 (AFP1), aflatoxin Q1 (AFQ1), aflatoxicol, and the highly toxic AFB1-exo-8,9-epoxide (AFBO) [25]. Unconjugated AFBO might form DNA adducts, driving genotoxic damage [26]. In our study, *CYP2A19* and *CYP3A29*, despite being key enzymes in AFB1-AFBO bioactivation known from studies in pigs [11, 12], did not show significantly enhanced mRNA expression. Another study examining pigs exposed to dietary AFB1 found no significant changes in the hepatic expression of *CYP2A19* and *CYP3A29*. This lack of change might be due to the already high baseline expression of these genes in the liver, limiting the ability to detect additional increases or decreases following exposure. The mentioned CYPs are described as pig-specific orthologs, and their limited expression may also reflect species-dependent differences in AFB1 metabolism. Consistent with our findings, Gerdmann et al*.* reported notable interspecies variation in AFB1 metabolism [27]. In a comparative study, hepatic microsomes from mice and monkeys generated the highest levels of AFBO, while human microsomes produced five times less AFBO than mice, further highlighting species specific metabolic profiles and detoxification capacities [28].

However, in the current porcine model, hepatic responses to dietary AFB1 exposure triggered a compensatory transcriptional shift affecting genes primarily involved in the regulation of endogenous metabolic pathways than in xenobiotic metabolism. This included the downregulation of *CYP2U1*, *CYP4V2*, *CYP7B1*, *CYP26A1* and *CYP51A1* which may collectively contribute to the accumulation or impaired clearance of primary and secondary metabolites such as fatty acids, cholesterol, and retinoids. Suppressed expression of *CYP2U1*, *CYP4V2* and *CYP7B1* suggests impaired arachidonic acid processing, fatty acid oxidation, and oxysterol clearance, contributing to hepatic lipid accumulation as observed in multiple species including humans, rats, mice, and ducks [29–31]. In humans, reduced *CYP51A1* may reflect disrupted cholesterol metabolism due to hydroxycholesterol buildup [32, 33], while in mice and calf, lower *CYP26A1* expression indicated retinoid accumulation, potentially impairing normal liver functioning [34, 35]. AFB1-induced lipid accumulation in hepatocytes, also observed at the histological level [36], might suppress expression of the aforementioned CYP enzymes through lipid-sensing pathways. Studies in ducklings indicated that excess lipid accumulation in liver can trigger transcriptional repression of CYP genes [37], likely as an adaptive mechanism to minimize metabolic overload. However, studies in rabbit hepatocytes indicate that lower CYP mRNA levels do not necessarily translate into reduced enzyme activity [38]. The transcriptional response to AFB1 exposure which subsequently might drive to a systematic metabolic disruption, is consistent with previous toxicological studies observed across several CYP families.

AFB1 intermediate conjugation is mediated by glutathione which is synthesized by glutathione synthetase (*GSS*) and is crucial for AFB1 epoxide detoxification. Subsequent elimination of AFBO-glutathione conjugates is facilitated via the ABCC2 protein [39, 40]. Reduced *GSS* and *ABCC2* expression observed in our study at low dietary AFB1 concentrations might limit glutathione conjugation of AFB1 epoxides and impair their clearance [41]. In this study, *SULT1E1* was notably repressed due to dietary AFB1 exposure. This finding points to a reduced estrogen sulfonation as *SULT1E1* acts as the key conjugation enzyme [42]. In a mice study, a sex-dependent effect of *SULT1E1* in the pathophysiology of liver injury affecting the protective role of estrogen was shown [43]. Since only female pigs were used in this study, results point to the fact that estrogen may play an important role in downstream signaling processes to effectively modulate AFB1 metabolism.

Among the antioxidant genes in our study, *GPX1* encoding glutathione peroxidase 1 was reduced following AFB1 exposure. GPX1 is crucial for reactive oxygen species (ROS) detoxification and redox balance [44]. A study in chicken primordial germ cells showed AFB1-driven ROS elevation resulted in mitochondrial damage, vacuolization, apoptosis, and upregulated *GPX1* [45]. In our model, however, oxidative overload might have overwhelmed *GPX1*, leading to its reduced expression. Another antioxidant enzyme PRDX4, a potent endoplasmic reticulum-based hydrogen peroxide scavenger, can become inactivated by excess H₂O₂ generated during AFB1 exposure, potentially explaining the reduced *PRDX4* expression [46, 47]. Notably, *NFE2L2* which showed a trend towards decreased expression following AFB1 exposure in our study, is a master regulator of oxidative defense, iron metabolism, and redox signaling driving ROS scavenging as well as glutathione and NADPH synthesis. AFB1 can disrupt *NFE2L2* activation, diminishing its downstream cytoprotective gene expression [48–50].

*ACOX1*, which catalyzes the rate-limiting step of peroxisomal fatty acid β-oxidation, was repressed in AFB1-exposed pigs. Since *ACOX1* inhibition promotes hepatic triglyceride accumulation [51], this downregulation parallels the lipid buildup linked to reduced CYP gene expression in our study, suggesting a compounded disruption of fatty acid metabolism.

In our experiment, ferroxidase ceruloplasmin *CP* expression was suppressed in response to AFB1 exposure. Similarly, previous studies in pigs exposed to mycotoxins reported reduced *CP* activity along with lower plasma ferric reducing antioxidant power, reflecting diminished non-protein antioxidant capacity [52–54]. Collectively, these reductions might suggest compromised ROS scavenging and impaired overall immunity in pigs exposed to AFB1. Additionally, C-reactive protein (CRP) is an acute-phase protein commonly regarded as a biomarker for inflammatory responses [55, 56]. In our study, decreased *CRP* levels observed in AFB1-treated pigs might indicate an impaired or altered acute-phase response due to toxin exposure.

### Medicinal herb-driven gene expression in liver

Andrographolide, the diterpenoid extract from kalmegh has already been shown to significantly upregulate CYP mRNA expression when synergistically combined with inducers like benzanthracene and β-naphthoflavone [57]. CYP2A19 and CYP3A29, key porcine AFB1 biotransformation enzymes [11, 12] were significantly increased in the kalmegh group. However, in a study using human hepatocytes, *CYP2A6*, the human orthologue of porcine *CYP2A19* was unaffected by kalmegh supplementation [58, 59]. This further emphasizes the species-specific differences in CYP enzyme responses. Kalmegh supplementation modulated lipid biotransformation and homeostasis by up-regulating *CYP2E1*, *CYP2U1*, *CYP4V2*, *CYP7B1*, *CYP26A1*, *SCD*, and *CP*. Most mammalian CYPs involved in xenobiotic metabolism have broad substrate specificity with implication on metabolic pathways [60]. The data indicated an increased hepatic turnover of lipids and reduced hepatic lipid accumulation with benefits for liver integrity [61–64]. For example, *SCD1* was shown to facilitate the conversion of excess hepatic lipids into monounsaturated fatty acids in murine or human hepatocytes, allowing for their safe storage within the organism [65]. Moreover, the upregulation of the other examined genes might indicate beneficial effects of kalmegh supplementation for liver integrity [66] as well as limiting cellular stress via higher glutathione conjugation [67, 68] and enhanced cellular protection [69]. Following Kalmegh supplementation, *SULT1C4* and *SULT1E1* were upregulated, indicating enhanced sulfonation activity. Given that andrographolide, the primary bioactive compound, is not a direct precursor to the highly anti-inflammatory derivative 14-deoxy-12(R)-sulfo andrographolide, this upregulation suggests an indirect mechanism. These findings might point to a key role of gut microbiota-dependent pathways involved in the bioavailability of andrographolide [70].

Dietary supplementation with milk thistle in pigs demonstrated a substantial overlap in affected hepatic gene expression profiles when compared to kalmegh. This suggests a concordant hepatic response between the two herbs, albeit with less pronounced overall effects observed for milk thistle. The differences in the transcriptional effects could be possibly due to milk thistle’s dose-dependent mode of action [71]. One of its secondary plant metabolites, silybin, which is known to boost the activity of a range of antioxidant enzymes such as superoxide dismutase plays a crucial role in neutralizing ROS [72]. Among genes upregulated by milk thistle supplementation, *GSTA1* is particularly important due to its role in regulating glutathione-xenobiotic conjugation. A previous study in rats demonstrated that milk thistle extract increases glutathione levels [73], and elevated *GSTA1* could enhance glutathione conjugation activity. This enhanced conjugation promotes xenobiotic detoxification and confer protection against oxidative liver damage, highlighting a potential protective mechanism of milk thistle supplementation [74].

Herbal supplementation with turmeric in pigs resulted in almost no differences in the hepatic expression profiles compared to the control and thus considerably deviated from the results with kalmegh or milk thistle [75, 76]. Additionally, curcumin, a key compound in turmeric, enhanced glutathione synthesis and directly scavenged ROS, highlighting its role as an antioxidant [77]. In chicken models, dietary curcumin has been shown to ameliorate AFB1 toxicity by modulating hepatic Phase I and Phase II enzymes, thereby providing hepatoprotection [78, 79]. However, in our study, turmeric supplementation solely reduced the expression of glutathione synthetase (*GSS*). This contrasts with earlier reports showing that curcumin activates the Keap1-Nrf2 pathway, which in turn increases *GSS* expression and promotes glutathione synthesis [80]. Moreover, curcumin exhibited concentration and time-dependent effects in murine macrophage cells. Low doses reduced oxidative stress and prevented cell death, while high doses increased ROS, which diminished with prolonged exposure [81]. The discrepancies suggest an experiment-dependent response, likely influenced by turmeric concentration and duration of exposure [82].

## Conclusions

This study reveals how aflatoxin and medicinal herb supplementation independently modulate hepatic gene expression related to biotransformation, detoxification, antioxidant defense, lipid metabolism and immune function. AFB1 at variable concentrations in feed formulations impairs CYP-mediated detoxification of reactive intermediates, undermining antioxidant defenses and glutathione conjugation. Among the herbs tested and their respective concentrations, kalmegh showed the strongest potential to restore hepatic adaptive responses, enhancing biotransformation capacity, toxin efflux, and antioxidant activity. Milk thistle elicited a moderate response, while the applied turmeric concentrations were less effective at the respective dose and duration tested in this experimental setting. By analyzing the effects of AFB1 and medicinal herbs in separate trials, this study provides essential baseline data that inform the design of more targeted and effective experiments with treatment combinations. This approach aligns with the principles of the 3Rs by refining experimental design and reducing the number of animals needed in future studies. Although the study investigates mRNA data, a functional validation of the results through protein assays or metabolite quantification is suggested. Incorporating medicinal herbs holds potential as a practical strategy to mitigate aflatoxin toxicity in livestock and may promote health and productivity in animals. These findings particularly support the use of kalmegh to counteract the hepatotoxic effects of aflatoxin-contaminated feed.

## Supplementary Information

Additional file 1. Standard feed mix. The file contains a table detailing the standard diet mix and chemical composition for EXP1 and EXP 2.

Additional file 2. Primer sequences. The file contains the primer sequences used for each target genes for both the experiments with forward and reverse sequences, primer size, melting temperature and amplicon size.

Additional file 3. Raw Ct data. The file contains raw Ct values for all samples in the study used for gene expression analysis.

## Data Availability

The datasets generated and analyzed during the current study are available in **Additional file 3**.
