# Supplementary material for: Transcriptional insights into aflatoxin B1 induced hepatotoxicity and comparative effects of medicinal herbs in pigs
Source: BMC Vet Res. 2026 Jan 15;22:53. doi: 10.1186/s12917-025-05270-1 (PMC12849138; doi:10.1186/s12917-025-05270-1)
Supplement: Supplementary file 1 — Additional file 1. Standard feed mix. The file contains a table detailing the standard diet mix and chemical composition for EXP1 and EXP 2. [file 12917_2025_5270_MOESM1_ESM.docx]

**Standard diet mix**

| **Feeding of piglets in Experiment 1** |  |
| --- | --- |
| The mash diet was offered twice daily at 07:00 and 15:00. The complete feed contained 63.6 % field-grown cereals (wheat, barley, maize, oats) plus purchased ingredients: soybean meal >46 % crude protein (9 %), processed soy protein (5 %), fish meal (4 %), fat (2 %) and a premix (4 %). All piglets received this basal diet. Group 1 also received 30 µg AFB1 kg⁻¹ body weight, Group 2 received 60 µg, Group 3 received 120 µg, and Group 4 served as the control. The toxin was given orally. Water was available ad libitum. Feed intake was recorded throughout the experiment. |  |
|  |  |
| **Feeding of piglets in Experiment 2** |  |
| The mash diet was offered twice a day, at 07:00 and 15:00. The complete feed contained 63.6 % field-grown cereals (wheat, barley, maize, oats) plus purchased ingredients: soybean meal ≥46 % crude protein (9 %), processed soy protein (5 %), fish meal (4 %), fat (2 %) and a premix (4 %). All piglets received this basal diet.  • Group 1 additionally received 15 mg kg⁻¹ body weight of andrographis (Andrographis paniculata). • Group 2 additionally received 45 mg kg⁻¹ body weight of silymarin (Silybum marianum). • Group 3 additionally received 45 mg kg⁻¹ body weight of curcumin (Curcuma longa). • Group 4 received only the basal diet. The herbs were mixed into the complete feed. Water was available ad libitum. Feed intake was recorded throughout the experiment. |  |
|  |  |
| **Diet components and composition for EXP 1 and EXP 2** | Value (%) |
| Wheat | **38.6** |
| Barley | **25** |
| Soybean meal > 46 % CP | **9** |
| Confectionery by-product | **12** |
| Processed soy protein (53 % CP) | **5** |
| Fish meal (60 % CP) | **4** |
| Premix***** | **4** |
| Feed-grade fat | **2** |
| Acidifier****** | **0.4** |
|  |  |
| * Premix (per kg): lysine 10.66 %, methionine 3.10 %, threonine 4.40 %, tryptophan 1.0 %, calcium 13 %, phosphorus 2.5 %, sodium 4.30 %, magnesium 2.5 %; vitamins A 400 000 IU, D 50 000 IU, E 3 250 mg, K 80 mg, B₁ 70 mg, B₂ 200 mg, niacin 650 mg, pantothenic acid 400 mg, B₆ 120 mg, B₁₂ 1 700 µg, biotin 8 000 µg, choline chloride 10 000 mg, folic acid 80 mg; trace elements Se 12 mg, Cu 2 500 mg, Mn 3 750 mg, Fe 4 000 mg, Zn 3 500 mg, I 70 mg; phytase 10 000 FTU g⁻¹; enzymes: endo-1,4-β-xylanase 12 200 U g⁻¹, endo-1,3(4)-β-glucanase 1 520 U g⁻¹; preservative citric acid 48.8 mg. ** Acidifier: formic acid 221 g kg⁻¹, propionic acid 60 g kg⁻¹, lactic acid 48 g kg⁻¹, acetic acid 40 g kg⁻¹, citric acid 20 g kg⁻¹, fumaric acid 10 g kg⁻¹, sorbic acid 10 g kg⁻¹. |  |
|  |  |
| **Chemical composition of the complete diet for EXP 1** | Value (%) |
| Dry matter | **88.92** |
| Crude protein | **18.21** |
| Crude fat | **2.82** |
| Crude fibre | **3.34** |
| Crude ash | **4.13** |
|  |  |
| **Chemical composition of the complete diet for EXP 2** | Value (% ) |
| Dry matter | **90.12** |
| Crude protein | **19.04** |
| Crude fat | **2.19** |
| Crude fibre | **3.35** |
| Crude ash | **5.18** |
